# Effects of crude oil on the somatic and immune development of juvenile threespine stickleback

**DOI:** 10.1371/journal.pone.0362078

**Published:** 2026-11-10

**Authors:** Kelly S. Ireland, Kathryn CA. Milligan-McClellan

**Affiliations:** 1 Department of Biological Sciences, University of Alaska Anchorage, Anchorage, Alaska, United States of America; 2 Department of Biology and Wildlife, University of Alaska Fairbanks, Fairbanks, Alaska, United States of America; 3 Department of Molecular and Cell Biology, University of Connecticut, Storrs, Connecticut, United States of America; Tanta University Faculty of Agriculture, EGYPT

## Abstract

Aquatic oil spills have profound effects on surrounding ecosystems, prompting significant investments in oil spill response efforts to remove oil from the environment as quickly as possible. Oil has immunotoxic effects and may be particularly harmful to larval and juvenile fish as it can cause a number of developmental defects and stunted growth. In spite of significant efforts to clean up oil, it is unclear whether larval and juvenile fish can recover from the effects of oil, and no work has been published on the effect crude oil has on developing threespine stickleback (*Gasterosteus aculeatus*) fish. Threespine stickleback are a ubiquitous sentinel species in the Northern Hemisphere and an important food source for many larger, economically valuable fish. As fish with fully marine, anadromous, and freshwater populations, stickleback are exposed to oil in a variety of aquatic environments. We aimed to understand fish recovery following removal of crude oil. Stickleback were exposed to Alaska North Slope crude oil and then were moved to water without crude oil for two weeks of recovery. Measurements of growth and immunity were taken before and after the recovery period. We found that crude oil effected different developmental pathways independently. Crude oil significantly impacted snout-vent length and eye diameter, but not swim bladder length or area, nor expression of *IL1B* and *TNF* genes. Stickleback fish were unable to recover from exposure after being transferred to clean water for two-weeks. This is the first study to examine the effect crude oil has on early stages of stickleback development and potential recovery. The results suggest larval and juvenile stickleback exposed to crude oil need longer than two-weeks to recover if they are able to recover at all.

## Introduction

Thousands of aquatic oil spills occur annually around the United States [1]. The spills range in size from small leaks (for example, localized, relatively short impact of less than a pint of engine oil spilled during an oil change in a personal-usage boat) to large spills with decades of impact over hundreds of miles of ocean and shoreline (for example, 134 million gallons of crude oil were released into the Gulf of Mexico during the Deepwater Horizon spill in 2010). Oil spills occur around the world and have occurred in both fresh and saline water bodies. Crude oil impacts fish at all life stages, including alteration of reproduction, mortality, cardiac function, development, immunity, and the microbiota [2–4]. Crude oil and polycyclic aromatic hydrocarbons (PAHs) found in crude oil also suppress the immunity of fish, like Southern flounder, Japanese flounder, and Chinook salmon [5–7]. For example, crude oil increases susceptibility to the pathogenic bacteria *Vibrio anguillarum* in Southern flounder (*Paralichthys lethostigma*) and Chinook salmon (*Oncorhynchus tshawytscha*) by suppressing the immune system, which leads to increased mortality [5,6,8]. Exposure to crude oil has acute and chronic effects on salmon fisheries, such as decreased marine survival and decreased reproductive output, which can impact spawning runs for subsequent salmon generations [9].

Oil exposure during early life is impactful as crude oil induces developmental defects [3,9,10]. Embryonic exposure to crude oil causes cardiac and craniofacial defects across a wide array of fish species including Atlantic haddock (*Melanogrammus aeglefinus*), polar cod (*Boreogadus saida*), and zebrafish (*Danio rerio*) [3,10–12]. Cardiotoxicity is both aryl hydrocarbon receptor independent and dependent [11,13]. Craniofacial effects of PAHs are induced by cardiotoxicity through a link between the growth of the basal neurocranium (ethmoid plate and trabeculae) and cardiac output [12]. Pink salmon juveniles exposed to oil have decreased survival in marine waters [9]. These defects can prevent fish from reaching maturity in the wild, and thus, overall population survival may be affected.

Recovery from crude oil exposure is species, time, and tissue dependent [9,14–16]. For example, juvenile pink salmon exposed to crude oil were less likely to survive to maturity and weighed less as juveniles than fish not exposed, but at maturity (after oil is removed) there were no differences in survival and weight [9]. In Australian Crimson-Spotted Rainbowfish, crude oil water accommodated fractions (WAFs, crude oil mixed in water prior to exposure to obtain a saturated hydrocarbon aqueous sample) caused an increase in citrate synthase, lactate dehydrogenase, and ethoxyresorufin-O-deethylase (EROD), but all levels returned to normal after a two-week recovery period [17]. Transcription of most genes in rainbow trout exposed to crude oil returned to normal after a 96 hour recovery period in gill tissue, but differential transcription was still observed in liver tissue after the recovery period, suggesting that recovery can be tissue dependent [15]. Immune suppression following exposure to bunker C crude oil was observed in Japanese flounder, but fish were able to recover after 1 week [16]. Unfortunately, most studies examining the recovery of fish from crude oil did not use statistical analysis that quantified the interaction between treatment and time [9,14–16]. Instead, the effect of treatment and time were analyzed individually, or the effect of treatment at each sampling time point was assessed independently of the previous time points [9,14–16].

Stickleback fish are important to the ecosystem as a ubiquitous sentinel species and food source in the Northern Hemisphere, existing as anadromous, oceanic, and freshwater populations [18]. Despite their importance it is unknown how crude oil affects stickleback. Anadromous stickleback could be exposed to marine oil spills as adults in the ocean or as spawning adults, embryos, larvae, and juveniles in tidally impacted estuaries and streams [19]. Freshwater populations of stickleback can be exposed to oil from oil pipeline spills, spills from other ground transport, or leaks from crude oil storage. Stickleback eggs do not float, so eggs can be exposed to dispersed oil in the water column or oil that sinks to the bottom. Stickleback eggs are also somewhat small (1.33–2.16 mm) [20]. This large surface area to volume ratio means there is more area for crude oil to adhere and for PAHs to pass through the chorion in relation to the eggs size, which may make stickleback embryos more susceptible to the effects of crude oil than fish with larger egg diameters, such as salmon (6 mm) [21,22].

The objectives of this study were to determine how growth and innate immunity in developing stickleback were affected by crude oil exposure, and whether fish were able to recover after they were moved to oil-free water. Larval fish were exposed to Alaska North Slope crude oil (a 10 mL mixture of stickleback embryonic media (SBEM) and 1.2 µL of crude oil added to 175 mL SBEM) for one week, between 7- and 14-days post fertilization (dpf), then moved to water free of oil for a two-week recovery period. Somatic measurements and immune gene expression were measured at 14- and 28-dpf. The interaction effect between treatment and time was assessed by statistical analysis that is more robust than previous studies, permitting inferences about the ability of the fish to recover from the oil exposure. This experiment was the first to characterize morphological and immune impacts and resiliency of threespine stickleback to crude oil and the first to use larval and juvenile stage threespine stickleback in an oil toxicology study.

## Materials and methods

### Fish husbandry

The research was carried out in strict accordance with the protocols approved by the University of Alaska Anchorage Institutional Animal Care and Use Committee (IACUC), including IACUC protocols #1302464–1 (Capture and Transportation), #1302466–2 (Husbandry), and #1363107–1 (Research Protocol for PAH and crude oil exposure). All staff who handled fish were trained on these protocols and IACUC and were listed as IACUC approved personnel. All euthanasia was performed with an overdose of tricaine MS-222 anesthesia per the IACUC approved protocols. Adult fish were collected from Bear Paw Lake in Houston, Alaska (61.61396, −149.75309) under Alaska Department of Fish and Game permit P-18–006 on October 5, 2018. These fish were acclimated in the lab for four months under a short light cycle of 10 hours of light per day, and then shifted to 20 hours of light per day to induce egg and viable sperm production. Embryos were generated by *in vitro* crosses on February 7, 2019. Sperm from two males were mixed and used to fertilize egg clutches from seven females. Eggs were fertilized, incubated for 2–4 hours to ensure viability, and then viable eggs were distributed randomly to six sterile polystyrene culture flasks (47 fish/flask) with filter caps that allow for gas exchange but no microbes to pass through (150 cm^2^, TPP Techno Plastic Products AG, Trasadingen, Switzerland) [23]. The number of eggs per flask (47 eggs/flask) was chosen so as to have an appropriate sample size for each sampling time point, 14- and 28-days post fertilization, and to prevent overcrowding in the flasks [24]. Each flask contained 175 ml of sterile 4 ppt instant ocean stickleback embryo medium (SBEM) made with Instant Ocean (Instant Ocean Spectrum Brands, Blacksburg, Virginia, United States) and ddH_2_O buffered with sodium bicarbonate and then filter sterilized with a 0.22 µM filter [23]. All fish were incubated at 18°C on a 20-hour light cycle in these flasks until 14 dpf with no circulation, addition, or removal of water except for at 7 dpf during the exposure protocol. At 8 dpf the flasks were flicked to release the remaining unhatched fish from their chorions.

Between 14 and 28 dpf the fish enter into the juvenile stage of development. At 14 dpf, fish not selected (via haphazard selection) for lethal sampling were moved into breeder nets within individual tanks for depuration on the same recirculating water that was free of crude oil, with an average salinity of 4.16 ppm and kept on a 20-hour light cycle at 16.3–17.6°C. Fish were fed brine shrimp and flake food (Golden Pearl Reef and Larval Fish flake food, Brine Shrimp Direct, Ogden, Utah, USA) daily. Fish health was monitored daily. Monitoring included recording mortality, removing dead fish, and observing status of fish development. In order to observe the effects of crude oil on fish development no humane endpoints were utilized for this research.

### Experimental design

To determine the effects of crude oil on threespine stickleback, at 7 dpf, 10 mL of SBEM were removed from each flask with a sterile pipet and an equal volume of oil or sterile SBEM was added. Flask treatment assignment was done with a random number generator. Randomly assigned flasks were exposed to oil at 7 dpf while fish were still in the larval stage of development. Fish were exposed to the exposure solutions during organogenesis [25], in the interest of observing sublethal effects since mortality is the main effect of crude oil on fish prior to organogenesis [26] and other studies show that the peak vulnerability to crude oil and single PAHs is during mid- to late-organogenesis [12,27–29]. Randomly assigned flasks were exposed to either 10 mL of sterile SBEM containing no crude oil (3 flasks; controls) or 10 mL of a crude oil/sterile SBEM mixture (3 flasks; treatment) (IACUC #1363107−1). Exposures were done by researchers who were not responsible for any measurements to keep treatments blinded. The oil mixture of Alaska North Slope Crude Oil was prepared by adding 1.2 µL of crude oil to 10 mL of SBEM in a sterile 15 mL falcon tube and then shaking the crude oil and water for 11.5 hours on a benchtop shaker at room temperature before addition to the flasks. Fish remained in flasks, with no additions of water or water changes, until fish were moved to tanks with oil-free water at 14 dpf. Samples and data were collected at both 14 and 28 dpf.

### Image and tissue collection

At 14 and 28 dpf, a subset of each flask or tank respectively (n = 12/flask or tank, except for one tank, where only 8 fish survived to 28 dpf so n = 8) were euthanized using greater than 250 mg/L MS-222 buffered with sodium bicarbonate (IACUC #1363107−1). The flask order for imaging was randomized by a random number generator. Standardized images of each fish were taken. After removing intestines for future sequencing analysis (DNA yield was deemed too low for analysis so no data is available), tissues from three individuals from the same tank were added to 200 µl Trizol with a 1:1 mix of 0.5 and 1.0 mm zirconia oxide beads in a RINO tube (Next Advance Inc. Troy, NY, USA), flash-frozen using liquid nitrogen, and stored at −80°C until RNA extraction. A timeline of the experiment and sample processing workflow is detailed in Fig 1. Taking measurements of fish at 14 dpf allowed us to observe the effects of crude oil on the development of fish after one week of exposure (between 7–14 dpf). We did measurements again at 28 dpf, two weeks after oil-exposed fish were removed from the oiled water, to determine whether recovery from crude oil was possible.

**Fig 1:**
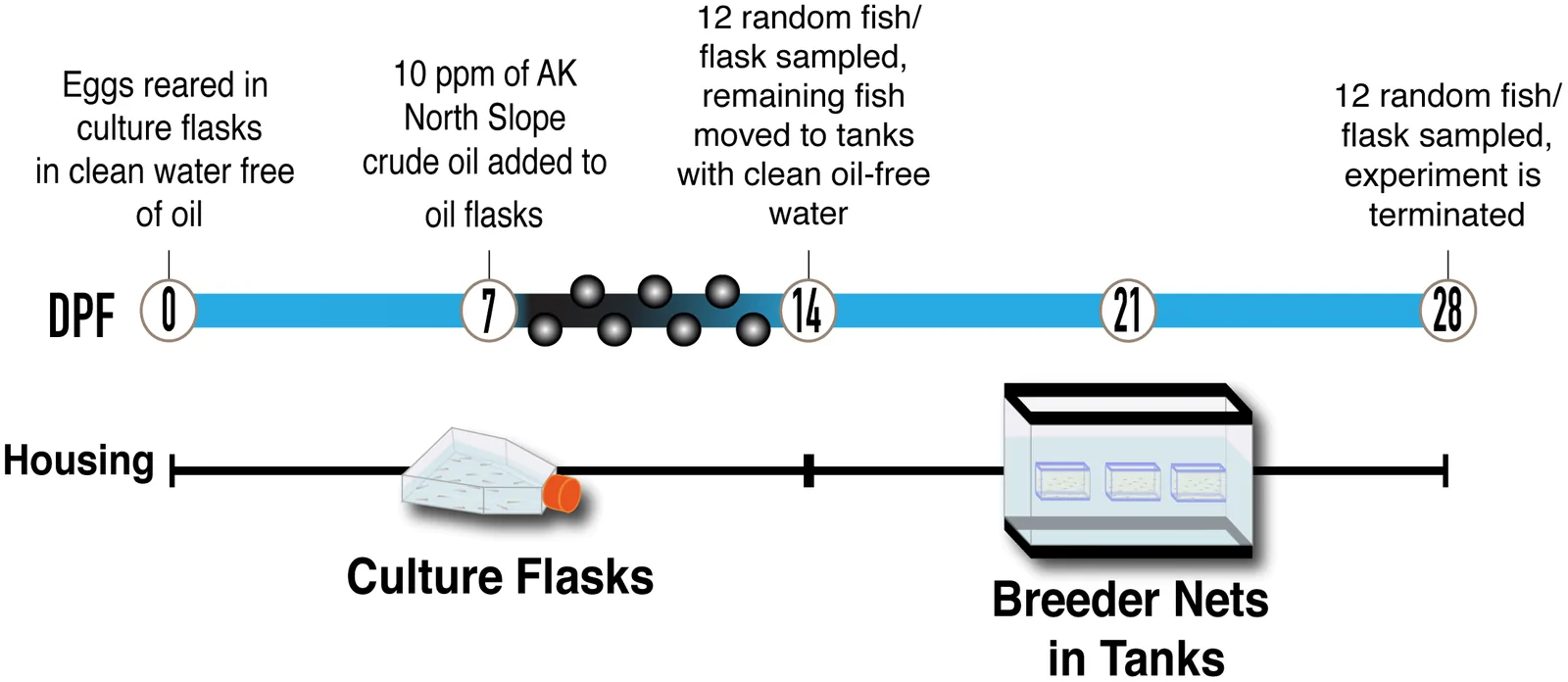
Experimental timeline and workflow this workflow was used to determine the effects of crude oil on juvenile threespine stickleback. Fish were reared in culture flasks from 1-14 dpf and in tanks from 14-28 dpf. Alaska North Slope crude oil or untreated water was added to randomly assigned flasks at seven dpf. Samples of 12 fish/flask were taken at 14 and 28 dpf. Fish were imaged, guts were dissected out (DNA yield too low for any analysis), and the whole bodies of three fish (from the same flask) were pooled for RNA isolation and qRT-PCR.

### Somatic development

Somatic development was measured using ImageJ software on images taken directly before gut dissections at 14 and 28 dpf on a dissecting scope. All photos from the same collection were taken with the same magnification, along with an image of a ruler for scale. Snout-vent length, eye diameter, swim bladder length, and swim bladder area were all measured from each fish (n = 12/flask except one control flask with n = 8 at 28 dpf, Fig 2). Each measurement was blinded for treatment and taken in triplicate by a single measurer and then averaged for analysis [30].

**Fig 2:**
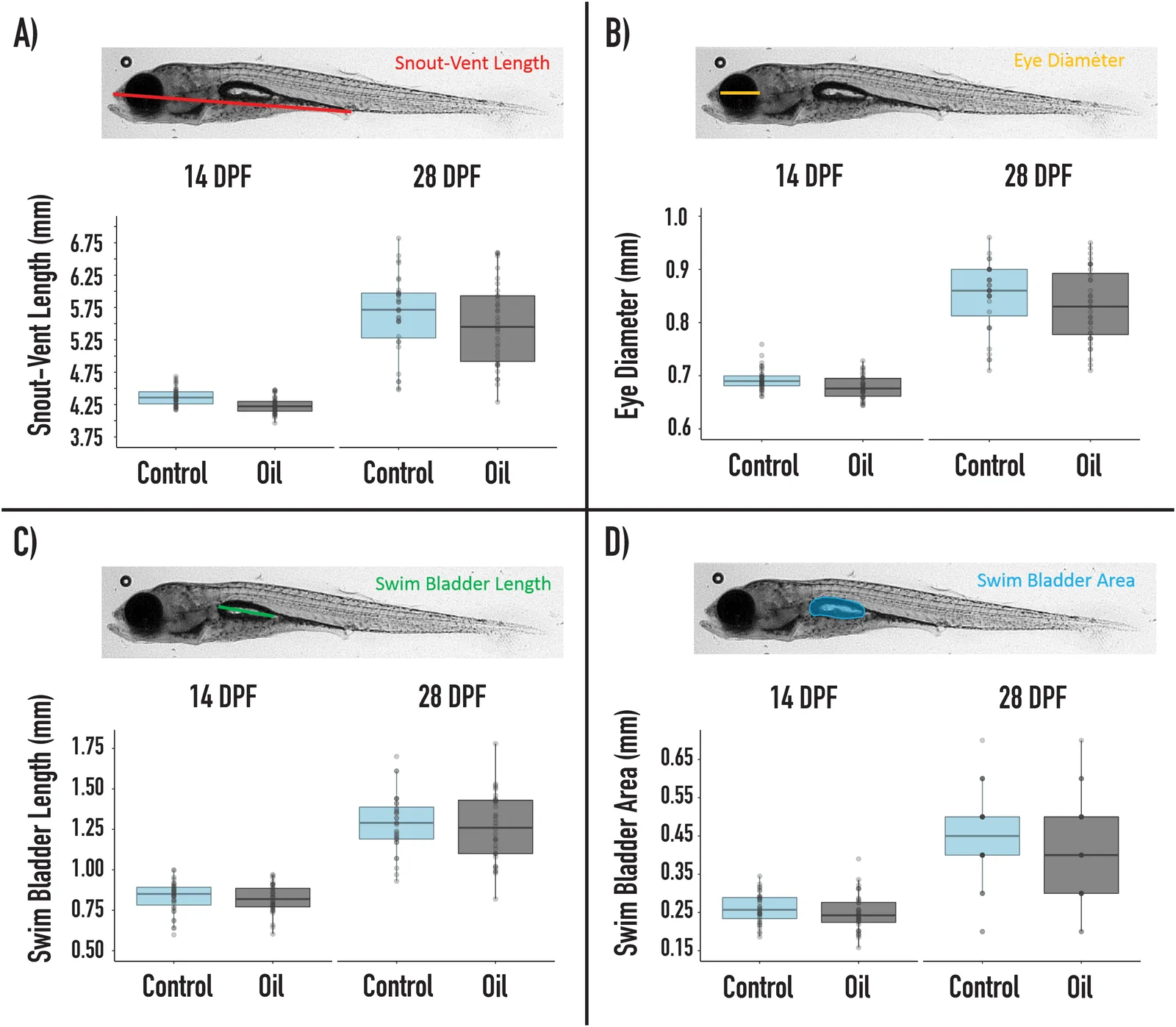
Somatic measurements measurements of somatic growth in oil-treated and untreated fish. Snout-vent length **(A)**, eye diameter **(B)**, swim bladder length **(C)**, and swim bladder area **(D)** measurements were made on 12 fish from each flask (except one 28 dpf flask that only had eight fish survive) at 14 and 28 dpf. There were three flasks/treatment. Boxplots represent control (blue) and oil-treated (black) fish with the median, 25th, and 75th quartiles, whiskers, and all outlying data. Dots represent measurements of individual fish.

### Immune gene expression

Expression levels of two innate immune system genes were measured using a well-established quantitative real-time polymerase chain reaction (qRT-PCR) protocol for samples taken at 14 and 28 dpf. A modified RNA isolation protocol using Trizol was used to isolate RNA from a pooled sample of 3 individuals with three pooled samples per flask at each time point [31]. Thawed samples were bead-beat on a Bead Mill24 homogenizer (Thermo Fisher Scientific, Waltham, Massachusetts, USA) before an additional 800 µl of Trizol was added, and the samples were flash-frozen again. The resulting homogenate was passed through a Qiashredder centrifuge column (Qiagen, Hilden, Germany) before two rounds of phase separation with chloroform in 2.0 phase lock gel tubes (QuantBio, Beverley, MA, USA) was performed. RNA was washed and eluted using the Qiagen RNAeasy Kit (Qiagen). RNA concentration, quality, and integrity were measured using a Qubit 4.0 Fluorometer, using the RNA High Sensitivity, Broad Range, and IQ Assay kits (Thermo Fisher Scientific). All RNA IQ scores were above 8, which indicates that the sample is mainly composed of larger RNA strands is of high quality. Average initial RNA concentration at 14 dpf was 67.11 ng/µl with a standard deviation of 28.59 ng/µl. Average initial RNA concentration at 28 dpf was 108.90 ng/µl with a standard deviation of 78.46 ng/µl. Initial RNA concentration did not significantly impact the expression of either gene. Working samples were created to a concentration of 50 ng/µl in RNase free water.

Each sample (n = 4 per flask or 12 per treatment at each time point) was analyzed in triplicate qRT-PCR reactions using the Luna Universal One-Step RT-qPCR kit (New England Bio Labs, Ipswich, MA, USA) according to the manufacturer’s protocol. Each reaction contained 10 µl reaction mix, 1 µl WarmStart reverse transcriptase, 0.8 µl forward primer (10 µM), 0.8 µl reverse primer (10 µM), 6.4 µl RNase free water, and 1 µl standardized RNA sample (50 ng). Relative normalized expression of two innate immune genes that promote inflammation, interleukin 1 beta (*IL1B*), and tumor necrosis factor alpha (*TNF*) were analyzed using established immune gene primers for stickleback [32]. The qRT-PCRs were run on a BioRad CFX96 Touch Real-Time PCR Detection System (Bio-Rad Laboratories, Hercules, CA, USA). The two genes of interest and two published, well-established stickleback reference genes, beta-2-microglobulin (*B2M*) and L13A ribosomal binding protein (*RPL13A*), were run in triplicate on the same plate for each sample [33]. The BioRad CFX Manager software version 2.1 [34] was used to calculate the relative normalized expression of *IL1B* and *TNF* against no template controls and the two reference genes. *IL1B* and *TNF* innate immune genes were chosen as stickleback lack adaptive immunity until later in their juvenile stage and because the innate immunity appears to be more affected by oil exposure in other fish than adaptive immunity [35–38].

### Statistical analysis

The proportion of dead fish in control and oil treatment groups from the start of exposure (7 dpf) to the end of the experiment (28 dpf) was compared using an N-1 chi-squared test to assess differences in mortality [39–41]. To assess the effects of oil exposure over time on both somatic responses and immune gene expression, a linear mixed-effects model of the following form was used:


$$\begin{array}{c} \text{y}=\alpha +{\text{a}}_{\text{i}}+\delta +\gamma +\delta *\gamma +\epsilon \end{array}$$ (1)

where y is the dependent variable (snout-vent length, eye diameter, swim bladder length, swim bladder area, *IL1B* or *TNF*), α is the overall intercept, a_i_ is a random effect of flask i (1–6), δ is the effect of treatment (oil or control), γ is the effect of time (28 dpf or 14 dpf), δ * γ is the interaction of treatment and time, and ε is a residual with mean 0 and variance σ^2^. Because of apparent differences in variances between 14 and 28 dpf, the model estimated separate variances by time. When the interaction between treatment and dpf was not significant, a reduced model with the interaction term was removed, and only the results from the additive model were reported. Snout-vent length and all other measurements could be allometrically correlated but were determined to not be correlated by a Pearson Correlation test (ED p = 0.975, SBL p = 0.926, SBA p = 0.902, *IL1B* p = 0.149, *TNF* p = 0.058) and therefore were analyzed separately. Data and the R markdown file can be found at https://github.com/kellysue94/effects_of_crude_oil_on_juvenile_threespine_stickleback_020719.

## Results

### Mortality

Crude oil can increase mortality in fish, but this varies by concentration, species, and life stage in which fish were exposed. To determine whether crude oil increases mortality in developing stickleback, deaths of fish were recorded daily and mortality rates between 7–28 dpf were compared between the control and oil treatments. Oil exposure did not significantly affect mortality rates (p = 0.9288, 5.08% mortality in control fish, 9 deaths, and 2.72% mortality in oil-treated fish, 3 deaths). Specific cause of death of individual fish was not determined.

### Somatic development

Length, eye diameter, and swim bladder structure are all impacted by crude oil in other fish [3,10,42–47]. The swim bladder Thus, we measured snout-vent-length - a proxy for total length – eye diameter, and swim bladder length and swim bladder area in stickleback one week after exposure (14 dpf) and after a two-week depuration (28 dpf) [30]. We expected all somatic measurements would be reduced at both 14- and 28- dpf in fish exposed to crude oil compared to control treated fish. Time caused a significant increase in all somatic measurements (Fig 2 and Table 1) as the fish grew from 14 to 28 dpf (p = 0.000 for all measurements). Both additive and interaction models were analyzed but given that the interaction term was not significant, only the additive effect of dpf and treatment is reported. Oil exposure significantly reduced snout-vent length (Fig 2A & Table 1, t = −4.14, df = 4, p = 0.0144) and eye diameter (Fig 2B, t = −2.86, df = 4, p = 0.0460) when time was held constant, meaning that snout-vent-length and eye diameter were smaller in oil exposed fish regardless if sampled at 14 or 28 dpf. Oil exposure did not significantly change swim bladder length (Fig 2C, t = 0.00132, df = 4, p = 0.999) and swim bladder area (Fig 2D, t = −0.500, p = 0.644). These results indicate that oil exposure significantly decreases snout-vent length and eye diameter, without recovery to control levels after a two-week depuration, but has no significant impact on swim bladder length or swim bladder area in this population of threespine stickleback, indicating that oil exposure effects development of the length and eyes, but not the swim bladder with these experimental conditions. No external pathological abnormalities such as lesions were observed in the control or treated fish.

**Table 1:** Somatic development and innate immune gene expression (relative normalized expression) summary statistics.

Measurement	dpf	Treatment	Mean	Standard Deviation	n
Snout-Vent Length	14	Control	4.371 mm	0.138 mm	36
		Oil	4.235 mm	0.128 mm	36
	28	Control	5.648 mm	0.605 mm	32
		Oil	5.486 mm	0.640 mm	36
Eye Diameter	14	Control	0.693 mm	0.021 mm	36
		Oil	0.678 mm	0.021 mm	36
	28	Control	0.848 mm	0.064 mm	32
		Oil	0.832 mm	0.068 mm	36
Swim Bladder Length	14	Control	0.808 mm	0.169 mm	36
		Oil	0.820 mm	0.087 mm	36
	28	Control	1.289 mm	0.178 mm	32
		Oil	1.259 mm	0.209 mm	36
Swim Bladder Area	14	Control	0.254 mm^2^	0.058 mm^2^	36
		Oil	0.249 mm^2^	0.048 mm^2^	36
	28	Control	0.447 mm^2^	0.114 mm^2^	32
		Oil	0.417 mm^2^	0.113 mm^2^	36
*IL1B*	14	Control	1.635	0.877	12 *
		Oil	2.242	1.587	12 *
	28	Control	4.716	4.104	10 *
		Oil	1.552	1.879	12 *
*TNF*	14	Control	1.790	1.134	12 *
		Oil	2.415	1.984	12 *
	28	Control	4.283	3.711	10 *
		Oil	1.394	1.579	12 *

Somatic measurements were made on 12 fish from each flask (except one 28 dpf control flask that only had eight fish survive) or 36 fish per treatment at each time point. Gene expression was measured on four pooled samples of three individuals from each flask (except one 28 dpf control flask that only had eight fish survive and only 2 pooled samples) or 12 pooled samples per treatment at each time point.

*3 fish in each pooled sample.

### Immune gene expression

Oil is immunotoxic to several fish species, but it is unknown whether oil exposure also causes immune suppression in stickleback. The expression of two innate immune genes involved in inflammation, interleukin 1 beta (*IL1B*) and tumor necrosis factor alpha (*TNF*), were measured.

There was no significant effect of treatment alone across the course of the experiment on the expression of *IL1B* (t = 2.490, df = 4, p = 0.1148) or *TNF* (t = 1.442, df = 4, p = 0.2226) (Fig 3). However, there was a significant positive effect of age on the expression of both genes (p = 0.000) and a significant negative effect of the interaction between treatment and dpf in both *IL1B* (t = −2.543, df = 39, p = 0.0000) and *TNF* (t = −4.532, df = 132, p = 0.0000) indicating that in control fish *IL1B* and *TNF* tended to be upregulated over time (from 14 to 28 dpf). However, in oil-treated fish, there was a downward trend in the expression of both genes from 14 to 28 dpf. The variance in the expression of both genes is decreased in the oil treated group. A Bartlett test indicated that the variance of both *IL1B* expression (14 dpf – p = 7.09x10^-4^,28 dpf – p = 1.66x10^-5^) and *TNF* expression (14 dpf – p = 1.33x10^-3^, 28 dpf – p = 2.91x10^-6^) in oil treated fish at 14 and 28 dpf was significantly reduced.

**Fig 3:**
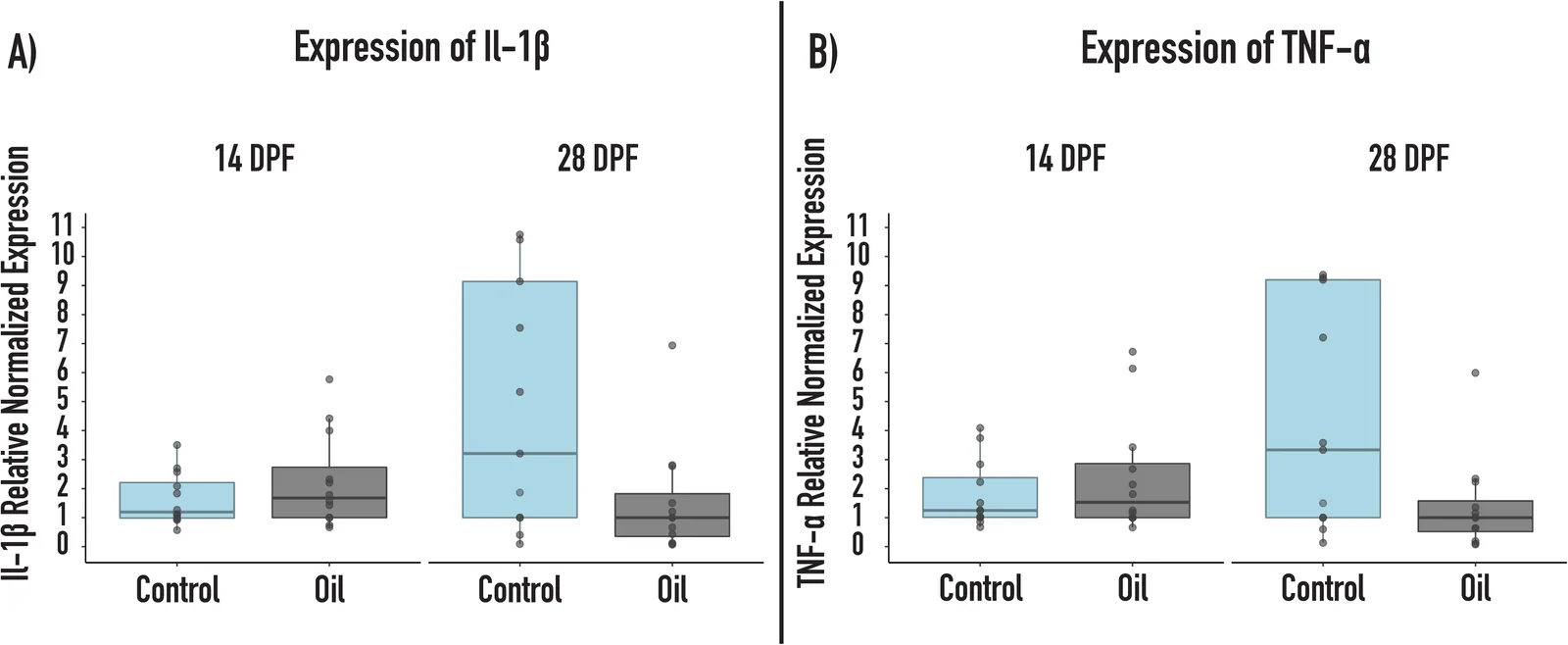
Immune Gene Expression Measurements of innate immune genes in oil-treated and untreated fish. Relative normalized expression of IL-1ß **(A)** and TNF **(B)** was measured on four pooled samples of three individuals from each flask (except one 28 dpf flask that only had two pooled samples) at 14 and 28 dpf. There were three flasks/treatment. Boxplots represent control (blue) and oil-treated (black) fish with the median, 25th, and 75th quartiles, whiskers, and all outlying data. Dots represent individual measurements.

## Discussion

### Mortality

Oil exposure did not increase mortality in larval and juvenile threespine stickleback. In this experiment, the oil was added to flasks at 7 dpf, just prior to hatching, which allowed fish to develop longer in their chorions before oil exposure. These results are similar to polar cod where mortality is not increased in fish exposed prior to hatching, but different than Southern flounder where mortality is increased. It should be noted that exposure concentration, method, and timing differed in these experiments [10,48,49]. However, juvenile Southern flounder and thinlip mullet (*Liza ramada*) exposed to oil post-hatch do not have increased mortality [6,50]. In Japanese flounder post-hatch increases in mortality only occur at higher concentrations of crude oil at 0.3 g/L [7], well above the amount of oil used in this study. Adult stickleback are also quite resilient to crude oil exposure, with a median tolerance, or concentration at which half the animals survive a given exposure, of 6.89 mg/liter [51], so it could be that developing stickleback are also fairly resilient to oil exposure.

While oil may not cause any increases in mortality ex-situ, where fish are in prime conditions, oil exposure could increase mortality in-situ, where conditions may not be ideal, and predators exist. Oil induced decreases in snout-vent length and eye diameter could impair visual acuity and swim performance, which can impact prey availability and predation [10]. There is also a trend of downregulation of innate immune genes, *IL1B* and *TNF*, which may make fish more susceptible to pathogens, thus increasing mortality in the wild.

### Somatic development

Snout-vent length and eye diameter are both significantly decreased in developing threespine stickleback exposed to crude oil. In Sheepshead Minnow, there were significant differences in length and weight when exposed to PAHs at as low as 50 mg ∑PAH/kg dry sediment [43]. In pink salmon, weight was lower in oil exposed fish at doses greater than 18 ppb before entering the marine environment, but there was no difference in size or weight when fish returned to their natal streams [9]. Crude oil exposure also decreases total length in Pacific herring [42]. Eye diameter is also reduced by oil exposure in polar cod [10]. Eye defects are also observed in bluefin tuna, yellowfin tuna, amberjack, Atlantic haddock, and more [3,44–46].

Threespine stickleback size was not able to recover to control levels during a two-week recovery period, suggesting if stickleback can recover, like salmon, it would take longer than two weeks. As mentioned in 4.1, the reduction of snout-vent length and eye diameter could impair visual acuity and swim performance, thus impacting prey availability and predation and ultimately survival [10].

The swim bladder is required for fish to rise in the water column, which is needed to capture prey and react to changes in the water environment. Crude oil can prevent swim bladder inflation in zebrafish [47] resulting in a decrease in swim bladder area. In this study, threespine stickleback swim bladders area and length were not affected by crude oil exposure. Stickleback and zebrafish do have quite different swim bladder physiologies which may be a potential reason why crude oil exposure causes non-inflation of the swim bladder in zebrafish and not stickleback. Zebrafish are physostomes (fish with a pneumatic duct to the swim bladder) and stickleback are physoclists (fish without a pneumatic duct) [52,53]. However, it is not known if threespine stickleback are transient physostomes or complete physoclists. Transient phystosomes are physoclists that during the larval stage have a pneumatic duct to the swim bladder that allows for initial inflation of the swim bladder, but then atrophies after inflation.

### Immune gene expression

PAHs, which are a component of crude oil, upregulate both *IL1B* and *TNF* transcription associated with inflammation in other fish species [7,36,54–56]. However, crude oil exposure did not have a significant effect on *IL1B* or *TNF* expression. Instead, there was a significant interaction of dpf and treatment on the expression of both, in which transcript levels of the genes tended to be lower over time in oil-treated fish and increased in the control fish. This is opposite to adult sheepshead minnow – where immune genes were downregulated after seven days of exposure to crude oil, but upregulated after 14 days of exposure [57].

Pathogen susceptibility and pathogen-induced mortality is increased in oil exposed fish [6–8]; thus, the decreased level of transcripts of both inflammatory genes over time may make threespine stickleback more susceptible to pathogens, increasing their mortality in situ. *IL1B* encodes for a cytokine that induces inflammation, fever, cell proliferation, differentiation, and pyroptotic cell death [58,59]. The activation of *IL1B* can occur from sterile urban particulate matter, not just pathogens [60]. *TNF* encodes for a cytokine that induces inflammation, cell proliferation, apoptosis, lipid metabolism, and coagulation [61,62]. *TNF* expression is also known to increase when exposed to aryl hydrocarbons receptor ligands like PAHs [63]. However, the expression of both genes was not changed by oil exposure alone.

These genes are not the only inflammation markers that are influenced by crude oil. PAH exposure increases macrophage activity in some species (like killfish) but decreased activity in other species (like carp) [35]. Splenic melano-macrophage centers also increase in number and size in sea trout and Gulf killfish when exposed to crude oil [64]. Thus, future experiments in stickleback should include other markers of inflammation to determine if other inflammatory pathways are impacted by crude oil alone. Experiments of threespine stickleback with combination oil and pathogen challenges should also be performed to determine the potential pathogenic susceptibility due to immunotoxic effects of crude oil.

Genes not associated with inflammation should also be examined to determine whether crude oil affects immunity in stickleback in other ways [5,6,8,9]. Primer sets for innate immune genes not associated with inflammation, such as the natural killer cell enhancing factor (*NKEF-β),* are limited for threespine stickleback. *NKEF-β* enhances the cytotoxicity of natural killer cells and protects cells against oxidative damage [65]. Nonspecific cytotoxic cells, which are similar to natural killer cells, are reduced in carp, oyster toadfish, and mummichog by PAH exposure [35]. Future studies could examine how crude oil affects *NKEF-β* expression or other nonspecific cytotoxic cells in threespine stickleback.

### Limitations

Fish oil toxicity studies typically expose subjects to high-energy water-accommodated fractions (HEWAFs) of crude oil [3,45,66]. HEWAFs are prepared by manual shaking of crude oil and water in a separatory funnel or by using a blender and blending the water and crude oil. The oil in this experiment was added to water and shaken on a benchtop orbital shaker before addition to the flasks. There was visible oil slicking and attachment to the sides of the tubes. The oil could potentially not have dispersed into the water as effectively as studies using HEWAFs. We also used polystyrene flasks; polystyrene acts as a sink for PAHs and sorbs all parent PAHs except fluorene and fluoranthene [67]. The intended crude oil exposure concentration in the water column was likely lower than intended. We did not perform analytical chemistry to confirm the concentration of oil as we had a very low volume of water remaining for each flask at the end of the experiment. In the future, gas chromatography mass spectrometry will be used to determine ∑PAH.

## Conclusions

Oil exposure in developing threespine stickleback has variable effects. Exposure does not impact the survival of developing stickleback, however exposure did significantly reduce eye diameter, which may impact visual acuity and swim performance and thus survival in the wild. Snout-vent length was also smaller in crude oil exposed stickleback, similar to salmon, which have reduced total length when exposed to oil [9]. The inflation of the stickleback swim bladder is not affected by oil exposure, unlike zebrafish [47]. Oil exposure alone also does not affect the expression of innate immune genes, *IL1B*, or *TNF*. However, fish exposed to oil have a downregulation in the expression of both genes from 14 to 28 dpf, whereas control fish have an upregulation between time points. These results are contrary to other studies in which crude oil and PAHs upregulate genes associated with inflammation [36,54,55,62]. Thus, developing threespine stickleback respond to crude oil exposure in some similar manners to other fish (reduced growth and no changes in survival), but differently in other ways (swim bladder inflation and no upregulation of genes associated with inflammation). Of the traits impacted by crude oil exposure alone, recovery was not achieved in the two-week depuration period. The trajectory of innate immune gene expression was also altered in oil exposed fish as expression increased over time in control fish but decreased over time in oil exposed fish. Taken together these results suggest that threespine stickleback need more than two-weeks to recover from the toxic effects of crude oil.
